# Consideration of psychosocial factors in workplace risk assessments: findings from a company survey in Germany

**DOI:** 10.1007/s00420-019-01416-5

**Published:** 2019-02-13

**Authors:** David Beck, Uwe Lenhardt

**Affiliations:** Bundesanstalt für Arbeitsschutz und Arbeitsmedizin, Berlin, Germany

**Keywords:** Workplace risk assessment, Psychosocial factors, Psychosocial work environment, Occupational safety and health, Company survey

## Abstract

**Purpose:**

Work-related psychosocial risks are an increasingly important issue in occupational safety and health (OSH) policy. In Germany, as in many other European countries, employers are legally required to carry out workplace risk assessments (WRAs) and to account for psychosocial factors when doing this. The aim of this study was to expand the still scarce and sketchy empirical evidence on the extent to which employers comply with these obligations, as well as on possible determinants of compliance behaviour.

**Methods:**

Survey data from 6500 German companies were used to calculate the prevalence of workplace risk assessments that include psychosocial factors. Furthermore, multinomial logistic regressions were performed to explore which company characteristics influence the chance of psychosocial risk assessment occurrence.

**Results:**

The prevalence of psychosocial risk assessments was 21%. Next to company size (OR = 5.7, 95% CI 3.0–11.0), availability of safety specialist assistance (OR = 3.5, 95% CI 2.6–4.6), availability of occupational health specialist assistance (OR = 3.4; 95% CI 2.6–4.4) and inspection by OSH authority (OR = 3.4, 95% CI 2.4–4.7) were the strongest predictors of psychosocial risk assessment occurrence. Smaller (but still significant) effect sizes were found for the level of knowledge about legal OSH requirements, training of managers in OSH, economic situation of the company, presence of a works council, positive view on the benefit of OSH, affiliation with the production sector and magnitude of psychosocial risks within the company.

**Conclusions:**

The study results indicate large deficiencies in the implementation of psychosocial risk assessments, especially for small companies. Findings suggest that enhancing companies’ utilisation of professional OSH experts and strengthening the advisory and control capacities of the OSH inspection authorities in the area of psychosocial risks would be beneficial for improving the current situation.

## Background

Stress at work is an important public health issue, as it contributes to the development of several widespread forms of physical and mental illness, such as coronary heart disease (Kivimäki et al. 2012) and depression (Madsen et al. 2017), burdening companies and society with high costs (European Agency for Safety and Health at Work 2014a). Work stress arises from an unfavourable psychosocial work environment, which includes aspects related to task layout, performance standards, work organisation, working time arrangements and social relations at work (Cox and Griffiths 2015). Over recent decades, profound economic, societal and political changes have led to a considerable (and still ongoing) transformation of the psychosocial work environment, making it increasingly relevant to the health of employees. An especially notable development is the expansion of the tertiary sector, which is paralleled by a steady growth in knowledge and interaction work. The proliferation of interconnected information and communication technology entails a tendency towards accelerating business and work processes as well as making jobs more mentally demanding. Driven by the ever-tightening global competition, many companies resort to frequent restructurings, ‘lean’ organisational practices, goal-oriented performance management methods or various forms of contingent work. As a result, many employees have to deal with an increase in flexibility and mobility requirements, job-related uncertainty and work intensity (Kompier 2006; Korunka and Kubicek 2017).

For these reasons, psychosocial factors at work have attracted more and more attention in the field of occupational safety and health (OSH). At the international, European and national levels, a wide range of policies, including both legislative and non-legislative approaches, have been developed by various stakeholders with the aim of improving psychosocial risk management within companies (European Foundation for the Improvement of Living and Working Conditions and European Agency for Safety and Health at Work 2014a, b; Leka et al. 2015). However, some concerns have been raised over the capability of these initiatives to achieve the intended improvements (Langenhan et al. 2013).

The degree to which psychosocial risks is included in the workplace risk assessment (WRA) procedure is an appropriate indicator for the effectiveness of the aforementioned policies, as the WRA is widely considered to be the core element of OSH management. The obligation to perform WRAs was introduced into OSH legislation in 1989 through the European Framework Directive on Safety and Health at Work (Council of the European Communities 1989). Since then, the related provisions have been transposed into national regulatory frameworks by all EU member states, making it mandatory for employers to determine the necessary occupational health and safety measures by carrying out an assessment of the risks the workers are exposed to at work. In doing so, all sources of risks, including psychosocial factors, should be considered. If psychosocial risks (e.g., excessive time pressure, conflicting demands, low job control, monotonous work, long/irregular working hours, lack of support from supervisors or colleagues, or violent threats from customers) were identified, measures must be taken to eliminate or minimise these risks as far as reasonably possible, and the measures taken should be reviewed for their effectiveness. Moreover, the results of the assessment, the measures derived and the evaluation of these measures must be documented (Beck et al. 2014; European Agency for Safety and Health at Work 2014b; Health and Safety Executive 2007).

Empirical information on how many companies account for psychosocial factors when performing WRAs is rather scarce, as very few countries collect or report such data. According to Denmark’s National Research Centre for the Working Environment, 65% of Danish companies surveyed psychological work environment factors in the 3 years prior to 2014 (Det Nationale Forskningcenter for Arbejdsmiljø 2017). In a Finnish study carried out in 2009, 82% of the surveyed managers and 63% of the surveyed workers’ representatives indicated that mental stress factors are considered in their companies’ WRAs (Niskanen et al. 2009). Figures available from two other countries are markedly lower: in the Netherlands, consideration of ‘work pressure’ (‘werkdruk’) in the context of WRAs occurred in 35% of the establishments concerned (Inspectie 2014), while 29% of French companies that had drawn up the mandatory risk assessment document had included psychosocial risks therein (Amira 2016). Apart from their highly limited geographical coverage, these data are also not very detailed; variations of psychosocial risk assessment prevalence according to company size and economic sector are the only empirical information provided. Other survey studies that touch on this issue are restricted to very specific study populations, such as workers’ representatives (Ahlers 2017), and are therefore of limited generalisability. The European Survey of Enterprises on New and Emerging Risks (ESENER) (European Agency for Safety and Health at Work 2016) cannot compensate for the shortcomings of the available national data, as it does not specifically refer to the inclusion of psychosocial factors in the WRA procedure legally prescribed by the EU Framework Directive on Safety and Health at Work.

The aim of the study reported in this article was to reduce the aforementioned knowledge gaps by (a) determining, on a representative basis, the prevalence of psychosocial risk assessments among companies in a large and highly developed economy (Germany), (b) estimating the quality of psychosocial risk assessments as reflected by their adherence to legally defined procedural requirements, and (c) exploring organisational factors that may impact the likelihood of a psychosocial risk assessment.

For this study, the case of Germany was chosen. In view of its economic significance and political clout, Germany is an interesting example both within the EU and globally. This makes figures from Germany a valuable contribution, since they provide an important point of reference for comparative research despite any national variations relating to legislation or institutional arrangements.

## Methods

### Data source

This study draws on survey data collected from a disproportionate stratified random sample of 6500 companies with at least one employee in mid-2015 as part of the evaluation of the German Joint Occupational Safety and Health Strategy (Gemeinsame Deutsche Arbeitsschutzstrategie—GDA). The target persons (i.e., the highest-ranking company members with responsibilities in OSH coordination) responded to a questionnaire, administered by computer-assisted telephone interview (CATI), on a wide range of safety and health topics, including several aspects of the WRA. Of the persons interviewed, 46% were company owners or managing directors, 23% were safety engineers and 31% were other company members (mostly managers) with responsibilities in OSH coordination. Even though field work was carried out according to generally accepted procedural standards, the net response rate did not exceed 15% (which will be discussed in the “Strengths and limitations” section of this article). The data were weighted to obtain a sample that is representative with respect to company size, sector and region. A more detailed description of the survey methodology (including the questionnaire) can be found in Sleik et al. (2015).

## Variables

### Psychosocial risk assessment

The basic questionnaire item concerning the WRA was “Are risk assessments being carried out at the workplaces in your company (yes; no; do not know; no answer (n/a))?” If WRAs were confirmed, respondents were to indicate whether the results of WRAs are being documented (yes; no; partly; do not know; n/a), whether needs for improvements had been identified in the most recent WRA (yes; no; do not know; n/a) and, if so, whether measures had been taken to realise the necessary improvements (yes; no; not yet but projected; do not know; n/a). If measures were reported, the respondents were asked whether the effectiveness of these measures was checked at a later date (yes; no; not yet but projected; partly; do not know; n/a). The scope of the WRAs (if any) was measured by two questions. First, the respondents were asked which of the following aspects of work were being routinely examined in this context (yes; no; do not know; n/a): “(A) Layout of the workplace”; “(B) Physical work environment”; “(C) Work equipment”; “(D) Working time arrangements”; “(E) Work organisation”; “(F) Social relations (between colleagues, between workers and superiors, between workers and customers)”. The second question referred to the kind of hazards that were considered in the company’s WRA (yes; no; not applicable, do not know; n/a): “(A) Hazards related to physical inactivity at the job”; “(B) Hazards related to the physical work environment, e.g., noise, heat, cold, dust”; “(C) Hazards related to hard physical work, e.g., carrying heavy loads, unfavourable postures”; “(D) Hazardous working equipment or machinery”; “(E) Hazards related to handling chemical or biological substances”; “(F) Hazards related to psychosocial workload”.

Based on the answers to these questions, different patterns of WRA implementation were determined as composite dependent variables for further analysis. According to the focus of the study, which was on psychosocial factors in the context of WRAs, a distinction was made between the following three categories:

- *Pattern A—Inactive with regard to WRA* Carrying out WRAs was not confirmed (response categories ‘no’, ‘do not know’ or ‘n/a’).
- *Pattern B—WRA without consideration of psychosocial factors* Carrying out WRAs was confirmed (response category ‘yes’), but consideration of hazards related to psychosocial factors was not (response categories ‘no’, ‘not applicable’, ‘do not know’ or ‘n/a’).
- *Pattern C—WRA considering psychosocial factors* Both implementation of WRAs and consideration of psychosocial factors were confirmed (response category ‘yes’).

Taking psychosocial risks into account is certainly an indispensable element of a WRA but does not necessarily mean that a WRA is fully consistent with legal demands. Therefore, the study further sought to capture those cases of psychosocial risk assessment that also met the other essential requirements of a WRA in a fairly comprehensive manner (the “cream of the crop”). Such cases could be defined using companies’ answers to several survey items on WRA, since these items closely correspond to the relevant legal provisions. Accordingly, pattern C from above was further divided into two subcategories:

- *Pattern C1—Incomplete WRA considering psychosocial factors* Both implementation of WRAs and consideration of psychosocial factors were confirmed, but fulfilment of one or more of the following requirements was not (response categories ‘no’‚ ‘do not know’ or ‘n/a’): (1) WRA documented; (2) measures taken if needs for improvements were identified; (3) measures checked for effectiveness if measures were taken; (4) all of the work aspects mentioned above were taken into account.
- *Pattern C2—Complete WRA considering psychosocial factors* All of the aforementioned questions were affirmatively answered.

For additional analyses, two more items from the questionnaire were included. One was about the issue of employee involvement in the WRA (“Are the employees being interviewed in the context of WRAs about hazards and health problems attributable to their jobs (yes; no; partly; do not know; n/a)?”). The other item was concerning the estimated benefit of the WRA (“How do you rate the benefit of WRAs in terms of OSH improvements in your company (very high; rather high; rather low; very low; do not know; n/a)?”).

### Characteristics of companies

Several factors that have previously been discussed as affecting company OSH practices were covered in the GDA survey questionnaire and could therefore be included in the present study as independent variables. These were company size (Hasle and Limborg 2006), sector (van Stolk et al. 2012), economic situation (Filer and Golbe 2003), magnitude of OSH risks (European Agency for Safety and Health at Work 2012), employee representation (Walters and Nichols 2007), specialist OSH assistance (Hämäläinen et al. 2001), workplace inspections by OSH authorities (Ko et al. 2010), knowledge about legal requirements (Sczesny et al. 2014), OSH training of managers and supervisors (Colligan and Cohen 2004) and perceived economic benefit of OSH (Zwetsloot et al. 2010).

Company size was determined by the question “How many employees, approximately, are working in your company?” The information obtained was categorised as follows: 1–9 employees; 10–49 employees; 50–249 employees; ≥ 250 employees. Sector was measured in two ways: First by the question “Does your establishment belong to the public service sector (yes; no, private business; do not know; n/a)?” and, second, using a dichotomous categorisation of the companies’ branch affiliations (production/agriculture; services). The economic situation of the surveyed organisation was measured by one question: “How do you rate the current economic situation (public service: ‘budgetary situation’) of your company (public service: ‘of your establishment’) (good; satisfactory; bad; do not know; n/a)?” The magnitude of psychosocial risks in the company was determined using the first two response categories of the following item: “How many employees in your company are exposed to the hazards listed below (almost all; rather many; rather few; almost none; do not know; n/a)? (…) (F) Psychosocial risks related to dealing directly with difficult clients, e.g., dissatisfied customers or patients; (G) Psychosocial risks concerning time/performance pressure; (H) Hazards resulting from social relations at the workplace, e.g., conflicts among colleagues or with the leadership style?” Respondents were further asked about the presence of an employee representative body (“works council”) in their company (yes; no; do not know; n/a). When data analyses related to this variable were carried out, ‘5–9 employees’ was used as the lowest size category, as legal regulations on works councils in Germany do not apply to companies smaller than that. Interviewees were also asked to indicate if their company has contracted a safety specialist (yes; no; do not know; n/a). If this was the case or if the respondent himself was a safety engineer, the company was classified as employing safety specialist assistance (as required by law). Specialist assistance in occupational health was measured by the question “Do you have a contracted occupational physician (yes; no; do not know; n/a)?” Regarding inspections, the respondents were asked whether their company had been visited by a competent OSH authority or accident insurance organisation since January 2013 and whether psychosocial risk assessment had been a subject of that visit (yes; no; do not know; n/a). Respondents further rated their level of knowledge about legal OSH requirements (very high; rather high; rather low; very low; do not know; n/a), indicated whether the company’s managers and supervisors receive training in OSH (yes; no; do not know; n/a) and characterised the general view of the company’s management on the benefit of OSH for company success (response categories: OSH helps to reduce costs; OSH increases costs without having an equivalent benefit; OSH neither contributes to nor interferes with company success; do not know; n/a).

### Statistical analysis

Descriptive analyses of weighted data were carried out using the CSTABULATE procedure from the SPSS statistical software package 18.0 for Windows. In these analyses, only companies with valid responses (i.e., answer categories “do not know” and “n/a” not considered) were included. As item non-response rates are rather low (to the most part ranging from 0 to 4%, with only 4 out of 32 items showing rates between 4 and 11%), no major problems occurred from this.

Multinomial logistic regressions based on unweighted data were performed to determine odds ratios (ORs) for WRAs with and without consideration of psychosocial factors according to relevant company characteristics (see above). In this context, an OR indicates the chance that a subgroup of companies exhibits a certain type of WRA implementation rather than having no WRA at all, in relation to the chance found in the reference group. While the univariate analyses included the total sample (i.e., companies with at least one employee, to which the legal obligation to carry out WRAs apply), the regression analyses were restricted to companies with ≥ 5 employees, as for one variable included in the multivariate model (i.e., presence of a works council), no data had been collected from companies smaller than that. The function of the interviewee within the company (owner/managing director, safety engineer, other) was additionally included as an independent variable in the multivariate model to account for possible responder bias. For multivariate analyses, the NOMREG procedure from SPSS 18.0 was used.

## Results

Table 1 displays the frequencies (absolute unweighted numbers and weighted percentages) of company characteristics which have been treated as independent variables in this study. The weighted sample largely consists of small companies, establishments from the service sector, and private businesses. The predominant economic situations among the companies were good and satisfactory. Just over one-third of the companies reported one or more psychosocial hazards that affect a large part of their workforce. Approximately one out of six companies has an employee representative body, and specialist assistance in safety and in occupational health is available in 48% and 36% of the responding organisations, respectively. 12% of the respondents confirmed that during the two and a half years prior to the survey, there had been an inspection that involved the topic of psychosocial risk assessment. The level of personal knowledge about legal OSH requirements was mostly (70%) rated as very or rather high. Clearly less than half of the companies arrange for manager or supervisor training in OSH. The vast majority of the respondents characterised the management’s view on the contribution of OSH to company success as either positive or neutral, and only one out of ten respondents indicated decidedly critical management views emphasising high costs and low benefits.

**Table 1:** Statistical overview of sample characteristics

Variable	*n* (unw)	% (w) (95% CI)
Number of employees (*n* = 6500)^a^		
1–9	1690	69 (67–71)
10–49	1891	25 (24–27)
50–249	1838	5 (4–5)
≥ 250	1081	1 (1–1)
Sector (I) (*n* = 6500)^a^		
Production/agriculture	2001	23 (21–25)
Services	4499	77 (75–79)
Sector (II) (*n* = 6465)^a^		
Private	5198	92 (90–93)
Public	1267	8 (7–10)
Economic situation of the company (*n* = 6205)^a^		
Bad	499	6 (5–7)
Satisfactory or good	5706	94 (93–95)
Magnitude of psychosocial risks in the company (*n* = 6198)^a^		
No psychosocial risks	3305	63 (60–65)
One psychosocial risk (out of three)	1656	23 (21–25)
Two or three psychosocial risks	1237	14 (13–16)
Works council (*n* = 5552)^b^		
No	3002	84 (82–85)
Yes	2550	16 (15–18)
Safety specialist assistance (*n* = 6462)^a^		
No	1583	52 (49–54)
Yes	4879	48 (46–51)
Occupational health specialist assistance (*n* = 6467)^a^		
No	2197	64 (62–67)
Yes	4270	36 (33–38)
Inspection by OSH authority concerning psychosocial risk assessment (*n* = 5803)^a^		
No	4348	88 (87–90)
Yes	1455	12 (10–13)
Level of knowledge about legal requirements in OSH (*n* = 6440)^a^		
Very/rather low	1395	30 (28–33)
Very/rather high	5045	70 (67–72)
Training of managers concerning OSH (*n* = 6280)^a^		
No	2775	59 (57–62)
Yes	3505	41 (39–44)
General view on the benefit of OSH (*n* = 6161)^a^		
OSH neither contributes to nor interferes with company success	2049	44 (41–47)
OSH increases costs without having an equivalent benefit	530	11 (10–13)
OSH helps reducing costs	3582	45 (42–48)
Total sample (*N*)	6500	100

*n* number of valid responses, *unw* unweighted, *w* weighted, *CI* confidence interval

^a^Number of valid responses; number of invalid ʻdo not knowʼ and ʻn/aʼ responses = difference to 6500

^b^Number of valid responses (basis: companies with ≥ 5 employees (*N* = 5571); number of invalid ʻdo not knowʼ and ʻn/aʼ responses = difference to 5571

### Prevalence and quality of psychosocial risk assessments

As shown in Table 2, just over half of the companies (54%) were confirmed to carry out WRAs, the vast majority of which (82%) were being documented. If needs for improvements were identified in the context of the WRA, which was true for approximately half of the cases (48%), measures to realise these improvements were almost always taken (95%) but were checked for effectiveness considerably less often (57%). Therefore, about two out of five WRAs that had resulted in the identification of OSH problems had also run through all the subsequent procedural stages described in OSH law.

**Table 2:** Implementation of workplace risk assessments (WRA)

Variable	*n* (unw)	% (w) (95% CI)
WRA carried out (*n* = 6355)^a^		
No	1439	46 (44–49)
Yes	4916	54 (51–57)
Results of WRA documented (*n* = 4878)^b^		
No	256	14 (12–17)
Partly	96	4 (3–6)
Yes	4526	82 (79–84)
Needs for improvements identified (*n* = 4729)^b^		
No	1730	52 (49–56)
Yes	2999	48 (44–51)
Measures taken (*n* = 2991)^c^		
No	43	1 (1–2)
Not yet but scheduled	133	4 (2–5)
Yes	2815	95 (93–97)
Measures checked for effectiveness (*n* = 2783)^d^		
No	391	23 (19–27)
Not yet but scheduled	526	20 (17–24)
Partly	32	0 (0–1)
Yes	1834	57 (52–61)
Work aspects considered in WRA		
Layout of the workplace (*n* = 4839)^b^		
No	309	10 (8–12)
Yes	4530	90 (88–92)
Physical work environment (*n* = 4848)^b^		
No	279	7 (6–9)
Yes	4569	93 (91–94)
Work equipment (*n* = 4864)^b^		
No	226	7 (6–10)
Yes	4638	93 (90–94)
Working time arrangements (*n* = 4787)^b^		
No	2424	51 (48–55)
Yes	2363	49 (45–52)
Work organisation (*n* = 4830)^b^		
No	1015	23 (20–26)
Yes	3815	77 (74–80)
Social relations (*n* = 4777)^b^		
No	3018	64 (61–67)
Yes	1759	36 (33–39)
Risk factors considered in WRA		
Hazards related to physical inactivity at the job (*n* = 4811)^b^		
No	1721	49 (45–52)
Yes	3090	51 (48–55)
Hazards related to the physical work environment (*n* = 4867)^b^		
No	787	27 (24–30)
Yes	4080	73 (70–76)
Hazards related to hard physical work (*n* = 4865)^b^		
No	1049	32 (29–36)
Yes	3816	68 (64–71)
Hazardous working equipment and machinery (*n* = 4874)^b^		
No	997	29 (26–33)
Yes	3877	71 (67–74)
Hazardous substances (*n* = 4879)^b^		
No	1604	45 (42–49)
Yes	3275	55 (52–58)
Hazards related to psychosocial workload (*n* = 4829)^b^		
No	2106	58 (55–61)
Yes	2723	42 (39–45)
Employees interviewed in the context of WRA (*n* = 4830)^b^		
No	1099	27 (24–30)
Yes/partly	3731	73 (70–76)
Estimated benefit of WRA (*n* = 4868)^b^		
Very/rather low	1292	37 (34–40)
Very/rather high	3576	63 (60–66)
Total sample (*N*)	6500	100

^a^Number of valid responses; number of invalid ʻdo not knowʼ and ʻn/aʼ responses = difference to 6500

^b^Number of valid responses, if WRAs have been carried out (*N* = 4916); number of invalid ʻdo not knowʼ and ʻn/aʼ responses = difference to 4916

^c^Number of valid responses, if needs for improvements have been identified (*N* = 2999); number of invalid responses ʻdo not knowʼ and ʻn/aʼ = difference to 2999

^d^Number of valid responses, if measures have been taken (*N* = 2815); number of invalid ʻdo not knowʼ and ʻn/aʼ responses = difference to 2815

While examining the layout of workplaces, the physical work environment and the work equipment is virtually standard practice (and looking at aspects of work organisation is still very common) when WRAs are being carried out (90%, 93%, 93% and 77% of the WRAs, respectively), working time arrangements (49%) and social relations at work (36%) are under scrutiny in only a minority of the cases. Accordingly, about seven out of ten WRAs consider hazards related to hard physical work (68%), working equipment and machinery (71%), or the working environment (73%), but only 42% of the companies with a WRA reported that psychosocial hazards are considered as part of the WRA.

Thus, the survey indicates that most German companies either show no WRA activities at all (pattern A) or have implemented the WRA without considering psychosocial hazards (pattern B). Only one out of five companies (21%) accounts for psychosocial hazards when carrying out a WRA (pattern C). Most of these WRAs may be categorised as incomplete with regard to process and scope (C1). The proportion of companies with a WRA that not only includes psychosocial risks but also meets the other requirements for complete implementation (C2) is 5% (Table 3).

**Table 3:** WRA implementation patterns, by company characteristics

Variable	Pattern A			Pattern B			Pattern C			Pattern C1			Pattern C2		
	*n* (unw)	% (w)	95% CI	*n* (unw)	% (w)	95% CI	*n* (unw)	% (w)	95% CI	*n* (unw)	% (w)	95% CI	*n* (unw)	% (w)	95% CI
Number of employees (*n* = 6500)^a^															
1–9	922	57	(54–61)	470	28	(25–31)	298	15	(13–17)	214	11	(9–13)	84	4	(3–5)
10–49	491	29	(26–33)	729	38	(34–42)	671	33	(29–36)	500	26	(23–29)	171	7	(6–9)
50–249	145	9	(7–12)	693	38	(35–42)	1000	53	(49–56)	766	40	(37–43)	234	12	(10–15)
≥ 250	26	2	(1–3)	301	28	(24–31)	754	70	(67–74)	513	47	(43–51)	241	23	(20–27)
Sector (I) (*n* = 6500)^a^															
Production/agriculture	280	37	(32–42)	857	44	(40–49)	864	19	(16–23)	618	15	(13–18)	246	4	(3–5)
Services	1304	51	(48–54)	1336	27	(24–29)	1859	22	(20–25)	1375	16	(15–19)	484	6	(5–7)
Sector (II) (*n* = 6465)^a^															
Private	1407	49	(46–52)	1769	31	(28–33)	2022	20	(18–22)	1460	15	(14–17)	562	5	(4–6)
Public	168	32	(24–41)	413	32	(25–40)	686	36	(29–44)	522	28	(22–35)	164	8	(5–13)
Economic situation of the company (*n* = 6205)^a^															
Bad	119	59	(47–70)	168	26	(17–38)	212	15	(10–22)	163	11	(7–17)	49	4	(2–9)
Satisfactory or good	1409	47	(45–50)	1930	31	(29–34)	2367	22	(20–24)	1719	16	(15–18)	648	5	(4–6)
Magnitude of psychosocial risks in the company (*n* = 6198)^a^															
No psychosocial risks	956	50	(47–53)	1228	34	(31–37)	1121	16	(14–19)	784	12	(11–15)	337	4	(3–5)
One psychosocial risk (out of three)	347	43	(38–48)	520	30	(25–35)	789	27	(23–32)	596	19	(16–24)	193	8	(6–11)
Two or three psychosocial risks (out of three)	226	45	(38–52)	323	20	(15–25)	688	35	(30–41)	516	27	(22–33)	172	8	(5–11)
Works council (*n* = 5552)^b^															
No	819	40	(37–44)	1145	36	(33–39)	1038	24	(21–27)	755	18	(15–20)	283	6	(5–8)
Yes	170	15	(12–20)	829	37	(32–42)	1551	48	(43–53)	1136	37	(33–41)	415	11	(9–14)
Safety specialist assistance (*n* = 6462)^a^															
No	972	66	(63–70)	395	24	(21–27)	216	10	(8–13)	177	8	(6–10)	39	2	(1–3)
Yes	584	27	(24–31)	1791	39	(36–42)	2504	34	(31–37)	1816	25	(23–28)	688	9	(7–11)
Occupational health specialist assistance (*n* = 6467)^a^															
No	1182	62	(59–66)	645	25	(22–28)	370	13	(11–15)	280	10	(8–12)	90	3	(2–4)
Yes	386	21	(18–25)	1536	41	(38–45)	2348	38	(34–41)	1708	28	(25–31)	640	10	(8–12)
Inspection by OSH authority concerning psychosocial risk assessment (*n* = 5803)^a^															
No	1304	52	(49–55)	1609	30	(28–33)	1435	18	(16–20)	1094	14	(12–16)	341	4	(3–5)
Yes	78	20	(14–28)	328	30	(24–37)	1049	50	(43–56)	716	33	(28–39)	333	17	(12–22)
Level of knowledge about legal requirements in OSH (*n* = 6440)^a^															
Very/rather low	641	67	(63–72)	440	23	(19–27)	314	10	(7–12)	261	9	(7–11)	53	1	(1–2)
Very/rather high	917	39	(36–42)	1732	34	(31–37)	2396	27	(25–30)	1722	20	(18–22)	674	7	(6–9)
Training of managers concerning OSH (*n* = 6280)^a^															
No	1087	61	(57–64)	915	25	(22–28)	773	14	(12–17)	641	11	(9–14)	132	3	(2–4)
Yes	422	28	(25–32)	1210	39	(36–43)	1873	33	(30–36)	1289	24	(21–27)	584	9	(8–11)
General view on the benefit of OSH (*n* = 6161)^a^															
OSH neither contributes to nor interferes with company success	724	53	(50–58)	712	32	(28–36)	613	15	(12–17)	506	12	(10–14)	107	3	(2–4)
OSH increases costs without having an equivalent benefit	141	45	(37–54)	203	37	(29–46)	186	18	(13–24)	141	14	(10–19)	45	4	(2–8)
OSH helps to reduce costs	612	41	(37–45)	1160	29	(26–33)	1810	30	(27–33)	1261	22	(19–25)	549	8	(6–10)
Total	1584	48	(45–50)	2193	31	(29–33)	2723	21	(20–24)	1993	16	(15–18)	730	5	(5–6)

^a,b^See Table 1

As seen in Table 3, there are several differences in WRA implementation between subgroups of the sample. Whereas total avoidance of a WRA (pattern A) is the predominant practice (57%) among micro-companies (1–9 employees) and is still fairly common (29%) among establishments with 10–49 employees, it is rarely observed in mid-sized and large companies. WRAs considering psychosocial factors (pattern C) are much more prevalent in large companies (70%) than in micro-enterprises (15%). The prevalence of psychosocial risk assessments (pattern C) is also distinctly higher in companies with the following characteristics: the presence of a works council (48%; no works council: 24%), an affiliation with the public sector (36%; private: 20%), availability of safety and occupational health specialist assistance (34% vs. 10%, and 38% vs. 13%), having large parts of the workforce affected by one or more psychosocial risks (27% and 35% vs. 16%), training managers/superiors in OSH (33% vs. 14%), having a high level of knowledge about legal OSH requirements (27% vs. 10%), having had a recent inspection by OSH authorities that involved discussion of psychosocial risk assessment (50% vs. 18%), and having a positive view on the economic benefits of OSH (30%; neutral: 15%; negative: 18%). For WRA pattern B, group differences are generally less pronounced, with the production (44%) vs. services (27%) difference being the most notable exception.

The observation that most of the WRAs that consider psychosocial hazards lack one or more other elements of complete implementation (C2 < C1) was made across all subgroups of the sample. Completely implemented psychosocial risk assessments (C2) were reported most frequently (> 10%) by large and medium-sized companies, by companies with a works council and by companies that have been inspected by OSH authorities.

The results further show that interviewing employees about work-related hazards and health problems is more common in companies that consider psychosocial factors in the WRA (C1/C2 > B), especially in companies where all work aspects are being examined and all process requirements fulfilled as well (C2 > C1). Furthermore, the percentage of companies rating the benefit of the WRA as very or rather high increases from 57% among pattern B to 82% among pattern C2 (Table 4).

**Table 4:** Employee involvement in WRA and management view on the benefit of WRA, by WRA implementation patterns

	Pattern B			Pattern C1			Pattern C2		
	*n* (unw)	% (w)	95% CI	*n* (unw)	% (w)	95% CI	*n* (unw)	% (w)	95% CI
Employees have been interviewed in the context of WRA (*n* = 4830)^a^									
Yes/partly	1463	68	(63–72)	1603	78	(73–82)	665	88	(79–94)
No	678	32	(29–37)	364	22	(18–27)	57	12	(6–21)
Evaluation of benefit of WRA (n = 4868)^a^									
Very/rather high	1444	57	(53–62)	1497	67	(61–72)	635	82	(75–88)
Very/rather low	720	43	(38–47)	479	33	(28–39)	93	18	(12–25)

^a^Number of valid responses (basis: companies which have carried out WRAs (*N* = 4916)); number of invalid ʻdo not knowʼ and ʻn/aʼ responses = difference to 4916

### Predictors of implementation

According to the results of the multivariate analysis presented in Table 5, the implementation of the WRA, especially a WRA that incorporates psychosocial factors, is strongly associated with company size. The chance of a WRA being carried out is five (OR = 4.8, 95% CI 2.5–9.1 for pattern B) and six (OR = 5.7, 95% CI 3.0–11.0 for pattern C) times higher in large companies (≥ 249 employees) than in small ones (5–9 employees).

**Table 5:** Results of the multinomial logistic regression analysis

Variable^a^	WRA Pattern B			WRA Pattern C		
	OR	95% CI	*p*	OR	95% CI	*p*
Number of employees						
5–9 (Ref.)	1.0			1.0		
10–49	1.4	1.1–1.8	0.009	1.5	1.1–2.1	0.006
50–249	2.7	1.9–3.9	0.000	3.1	2.1–4.6	0.000
> 249	4.8	2.5–9.1	0.000	5.7	3.0–11.0	0.000
Sector (I)						
Services (Ref.)	1.0			1.0		
Production/agriculture	2.6	2.0–3.3	0.000	1.5	1.2–2.0	0.002
Economic situation of the company						
Bad (Ref.)	1.0			1.0		
Satisfactory or good	1.4	0.9–2.1	0.094	1.9	1.2–2.8	0.004
Magnitude of psychosocial risks in the company						
No psychosocial risks (Ref.)	1.0			1.0		
One psychosocial risk (out of three)	1.0	0.8–1.3	0.919	1.4	1.1–1.9	0.010
Two or three psychosocial risks	0.8	0.6–1.0	0.076	1.4	1.0–1.8	0.045
Works council						
No (Ref.)				1.0		
Yes	1.5	1.1–2.0	0.023	1.8	1.3–2.5	0.000
Safety specialist assistance						
No (Ref.)	1.0			1.0		
Yes	2.7	2.1–3.5	0.000	3.5	2.6–4.6	0.000
Occupational health specialist assistance						
No (Ref.)	1.0			1.0		
Yes	2.5	2.0–3.2	0.000	3.4	2.6–4.4	0.000
Inspection by OSH authority concerning psychosocial risk assessment						
No	1.0			1.0		
Yes	1.2	0.8–1.6	0.351	3.4	2.4–4.7	0.000
Level of knowledge about legal requirements in OSH						
Very/rather low (Ref.)	1.0			1.0		
Very/rather high	1.9	1.5–2.4	0.000	2.9	2.2–3.7	0.000
Training of managers concerning OSH						
No (Ref.)	1.0			1.0		
Yes	2.0	1.6–2.5	0.000	2.7	2.2–3.4	0.000
General view on the benefit of OSH						
Neither contributes to nor interferes with company success (Ref.)	1.0			1.0		
Increases costs without having an equivalent benefit	1.1	0.8–1.5	0.713	1.0	0.7–1.4	0.903
Helps to reduce costs	1.2	1.0–1.5	0.059	1.7	1.4–2.2	0.000

*OR* odds ratio. *N* = 4271 companies with ≥ 5 employees; only valid responses; Nagelkerke’s Pseudo-*R*^*2*^ = 0.411

^a^The variables ‘Sector II’ and ‘Function of responder’ were ommitted from this table as they showed no significant effect in the multivariate model (see chapter ‘Predictors of implementation’)

After company size, the availability of safety specialist assistance (OR = 3.5, 95% CI 2.6–4.6), the availability of occupational health specialist assistance (OR = 3.4, 95% CI 2.6–4.4) and having had an inspection visit by OSH authorities (OR = 3.4, 95% CI 2.4–4.7) show the strongest effects on psychosocial risk assessment occurrence (= WRA pattern C), with ORs larger than 3. ORs between 2 and 3 (pattern C) were found for having a very/rather high level of knowledge about legal OSH requirements and for training managers/supervisors in OSH. Of the remaining predictor variables, the presence of a works council, being in a good or satisfactory economic situation, recognising OSH as helping to reduce costs, an affiliation of the company with the production sector, and a self-reported presence of psychosocial risks in the company show small to moderate associations (1 < OR < 2) with pattern C. However, being categorised as either a public or a private establishment (OR = 0.9, 95% CI 0.6–1.2) and the function of the responder within his company (safety engineer: OR = 1.2, 95% CI 0.8–1.6, and owner/managing director: OR = 0.9, 95% CI 0.7–1.2) have no significant effects in this regard (not presented in Table 5).

As far as WRA pattern B (i.e., psychosocial factors not included in the WRA) is concerned, the effect sizes of the predictor variables are generally smaller, with sector variable I (production vs. services) being the only exception (OR_pattern B_: 2.6, OR_pattern C_: 1.5). Furthermore, two variables that proved to be associated with WRA pattern C (i.e., presence of psychosocial risks in the company and having had an inspection visit concerning psychosocial risk assessments) show, for obvious reasons, no significant association with pattern B.

## Discussion

The results of this study indicate that in Germany, as in other European countries (Amira 2016; Inspectie 2014), the obligation to consider psychosocial factors when carrying out WRAs is still not put into practice by most companies. Even among large establishments with 250 or more employees, three out of ten report negatively in this regard. This study therefore confirms recent qualitative findings based on observations reported by German OSH consultants (Lenhardt 2017).

Several reasons for psychosocial risks being a particularly “wicked problem” that is more difficult to deal with than “traditional” OSH problems (such as accidents or noise) have been noted by Helbo Jespersen et al. (2016): among other things, psychosocial risks are characterised by unclear cause–effect relationships and rather uncertain solutions; they are largely determined by variable subjective perceptions of work situations; they closely relate to the core area of employer prerogatives, particularly the organisation of work; and attempts to tackle them easily engage opposing interests and entail challenges to the managers’ exercise of power. Altogether, these characteristics may explain, in large part, the generally low level of company activity regarding psychosocial risk assessments.

However, our data show that certain companies are less likely than others to account for psychosocial factors when assessing workplace risks. This is especially evident among small establishments, which have repeatedly been shown to have greater deficiencies in OSH performance for a variety of reasons (Walters and Wadsworth 2016a). In this context, previous research has emphasised that small companies operate under particularly volatile conditions, and management responsibilities are often concentrated on one person. As a result, these organisations not only have comparatively limited resources (in terms of personnel, time, money, skills and knowledge) to devote to seemingly “unproductive” activities such as OSH but are also more disinclined to use formalised systematic management approaches in this area (Champoux and Brun 2003; Hasle and Limborg 2006; Masi and Cagno 2015). Moreover, small companies were found to exhibit a stronger tendency towards trivialising, or even denying, work-related safety and health risks, which may be partly attributed to accidents being very rare events in individual small establishments (Hasle et al. 2009). It is important to consider, however, that many small businesses are used to handling problems in a very personal and highly pragmatic manner without deploying predefined and clearly structured procedures and protocols. This most likely applies to safety and health issues as well. Although very little research has been done on this subject (Pinder et al. 2016), it may therefore be assumed that especially small companies would rather resort to informal and implicit (but not necessarily ineffective) ad hoc practices of “assessing” and “managing” occupational risks (Beck et al. 2017). Such practices may remain, at least partly, undetected if survey respondents are asked about adhering to legally prescribed WRA procedures.

With so many companies being highly reluctant to address psychosocial risks because of their particularly “wicked character” mentioned above, it seems obvious that an external “push”, for instance that provided by inspection visits, is often needed to get things going. In fact, our study results suggest that being inspected by an OSH authority clearly makes a difference with regard to a company’s willingness to carry out psychosocial risk assessments. A recently published review presented some evidence in agreement with this finding but also concluded that the overall impact of inspections on psychosocial risk management is still rather limited for various reasons, such as unfavourable regulatory and labour market contexts, underdeveloped inspection strategies, lack of resources, insufficient training, or a low frequency of inspection visits (Weissbrodt and Giauque 2017). Some of these circumstances apply to Germany as well, although a number of efforts have been made in recent years to improve the situation (Beck et al. 2011; Ertel 2014). In Germany, as in most other developed countries, arranging for adequate staffing and providing OSH inspectors with the skills needed for effectively intervening in the area of psychosocial risk management still remain considerable challenges (Johnstone 2016).

Similar conclusions can be drawn with respect to OSH specialist assistance. To our knowledge, the present study was the first one to examine associations between the implementation of psychosocial risk assessments and the utilisation of safety engineers and occupational physicians, proving that both are strong predictors of a company’s capability to incorporate psychosocial risks into a WRA. However, it must be considered that according to our survey data, a great number of companies do not make any use of professional OSH consultants, or, if they do, are still quite often dealing with OSH specialists who have little expertise in psychosocial risk prevention and show rather low attention to that issue in their daily practice. The latter is certainly true for safety engineers (Hamacher et al. 2013; Leitão and Greiner 2017), whereas findings for occupational physicians are more mixed (Harber et al. 2010; Persechino et al. 2016). Overall, the potential benefits of OSH specialist assistance for promoting psychosocial risk assessments still seem far from being fully exploited.

Since psychosocial risks are closely connected to issues of management decisions and power relations at work, it may be assumed that worker representation is rather important for whether or not these risks are being systematically addressed within companies. Previous research from the Netherlands (Popma 2009) and Germany (Ahlers and Brussig 2005) found some evidence that corroborates this assumption. Our own study also revealed that psychosocial risk assessments are more likely to occur when works councils are in place, but the association, although statistically significant, was not as strong as might be expected considering the extensive co-determination rights of employee representative bodies in Germany. Apparently, the mere presence of works councils (which is a rare enough thing in most countries) does not make much of a difference after all in regard to psychosocial risk assessments. The decisive point here seems to be the workers’ representatives’ actual scope and ability to mobilise sufficient power resources and make full use of their statutory rights (Popma 2009), but such favourable circumstances are by no means the rule, as evidenced by a recent empirical study on determinants and patterns of workers’ representation in OSH (Walters and Wadsworth 2016b).

As mentioned above, the consideration of work-related psychosocial risks in the management of OSH—especially in WRAs—is mandatory for employers according to EU and national regulations (European Foundation for the Improvement of Living and Working Conditions and European Agency for Safety and Health at Work 2014a, b; Zoni and Lucchini 2012). Given that psychosocial risk assessment activity is associated with the company’s level of knowledge about the legal obligations in OSH, its low prevalence might be attributed to a general lack of information on what is required from companies by OSH law. At first glance, this interpretation seems to be at odds with GDA survey results showing that as many as 70% of company representatives describe their knowledge about legal OSH requirements as very or rather good. However, a more thorough examination of what managers know about OSH regulations presented a much less optimistic picture, especially for small and medium-sized enterprises (Sczesny et al. 2014). Judging from this, plain ignorance of OSH provisions among duty holders, in addition to methodological problems and issues of power, may still be a major obstacle to the implementation of psychosocial risk assessments. As our findings suggest, intensifying manager training in OSH, which is implemented in only two out of five companies, would be highly beneficial in this regard.

Over the years, there has been a recurrent debate on the relevance of the “business case” for promoting OSH management, and psychosocial risk management in particular, emphasising the need to complement regulatory approaches with strategies to increase companies’ understanding of the favourable effects preventive action may have on their economic performance (Leka et al. 2015). In principle, this idea is supported by our study, which found that the consideration of psychosocial factors in WRAs is associated with the management’s belief that OSH helps to reduce costs. However, the effect size is rather small, indicating that a generally positive management view on the economic benefits of OSH may not be a particularly important driver for psychosocial risk assessments. Quite astonishingly, this is also true for the magnitude of psychosocial risks within the company (as estimated by the surveyed company representatives). Even though the chance of psychosocial risk assessments being implemented is slightly elevated in workplaces where many or almost all employees are considered to be affected by at least one psychosocial risk factor, it is quite obvious that the presence of the problem, per se, has no strong implications for company actions. Therefore, additional facilitating circumstances must be present to induce substantial efforts in the development of psychosocial risk management at the company level.

Finally, two other study findings that may challenge, or at least qualify, some conventional wisdom about the conditions for managing psychosocial risks should be highlighted. One assumption is that dealing with these kind of risks is more relevant to the service economy than to the production sector, where the main emphasis is on material risk factors instead. However, our data suggest that both studied categories of WRAs—those confined to physical or chemical hazards as well as those including psychosocial factors—are more likely to be found in establishments from the production sector. Even though the effect was fairly small for the second WRA pattern, this indicates that considering psychosocial risk management to be primarily a service sector issue would be rather misleading.

Given that appropriately addressing the psychosocial work environment is a challenging task for most companies that could absorb a considerable amount of their organisational capacities, it might also seem natural to suppose that prosperous companies are clearly more inclined to take action in this field than those performing poorly in economic terms. Indeed, the present study revealed that the chance of psychosocial risk assessments occurring is higher when the company’s economic situation is rated as good or satisfactory. However, the established effect was not very large, indicating that the incorporation of psychosocial risks into OSH practice, although benefiting from a thriving business, is not a sheer “fair weather phenomenon”.

## Strengths and limitations

This study has several strengths. First, it is based on data from a comparatively large sample that is not only representative of German companies with regard to size, branch and region but also allows for differentiated subgroup analyses. Second, data were obtained from company managers or functionaries who are, due to their decision-making and coordinating responsibilities, likely to be better informed about the organisation’s preventive activities than ordinary employees are. Furthermore, this study, in contrast to previous survey-based research on psychosocial risk assessments, does not restrict itself to merely examining bivariate distributions, but analyses the independent influence of various organisational characteristics on psychosocial risk assessment occurrence in a multivariate model.

There are also some noteworthy limitations of this study. As mentioned in the “Methods” section, companies with less than five employees, which account for 929 cases, or 14% of the unweighted sample, had to be excluded from the multivariate analyses because of missing data for one of the explanatory variables (i.e., “works councils”). This raises the question as to whether the findings can be generalised to the entire sample. For testing purposes, we included very small companies (1–4 employees) in a repeated multinomial regression, assuming a zero prevalence of works councils in this group (which is, in view of the German regulations on works councils and high-quality data on workers’ representation in Germany (Ellguth and Kohaut 2018), quite realistic). In this rerun, all associations remained virtually unchanged (results not shown), indicating that the validity of the multivariate findings is not restricted to companies with ≥ 5 employees.

The response rate in the survey our study is based on was rather low, even lower than the typically low rate for most non-enforced business surveys (Rasmussen and Thimm 2009). One possible reason for this is that the particular subject of this survey (i.e., “safety and health”) normally might not attract as much of a company’s attention as other subjects that are more closely business related. Moreover, companies in Germany seem to be generally less willing to participate in such surveys than companies in most other European countries are, as comparative data from ESENER-2 (European Agency for Safety and Health at Work 2016) or the European Company Survey (European Foundation for the Improvement of Living and Working Conditions 2015) show.

The low response rate of the GDA survey may have resulted in substantial non-response bias. Unfortunately, we could not perform a detailed non-responder analysis, because the necessary data were unavailable to us. According to the technical report of the GDA survey, empirical information on non-responding companies based on sampling frame data is restricted to three characteristics (i.e., size, economic sector and region) that might be related to our outcome variable. Although small companies and establishments from the private services sector turned out to be clearly overrepresented among the non-participants, this should be of no consequence in terms of bias as it was compensated for by non-response adjustment of sample weights.

However, significant residual bias resulting from other factors cannot be ruled out. Theoretical considerations and empirical studies on the determinants of survey response have pointed out that the potential responders’ attitudes and behaviour with respect to the topic of a survey strongly influence their willingness to actually participate in it. The more they are interested in and committed to the topic and the better they perform (or think to perform) in the area covered by the topic, the higher their propensity to respond will be (Groves et al. 1992, 2004; van Goor and Verhage 1999). Based on this, it may be assumed that companies lacking awareness and activity in the field of OSH (including WRAs) were generally more likely to refuse participation in the GDA survey, which explicitly focussed on OSH matters. Therefore, a tendency towards overestimating the prevalence of psychosocial risk assessments resulting from non-response bias must be accounted for.

As our study is not only about prevalences but also about predictors of WRA activity, considering possible relational non-response bias is equally important as reflecting on distributional bias. Again, it must be noted that performing statistical analyses to test for non-response bias (relational or other) would have required data that are not available in the case of the GDA survey. Consequently, we are not able to verify in a methodologically sound manner, whether (or, if so, to which extent) our results are biased by non-response. However, there is a considerable body of evidence suggesting that survey non-response, while often inducing significant bias in univariate estimates, mostly does not affect the relationships between the variables under study, even if it is at high levels (Amaya and Presser 2017; Blair and Zinkhan 2006; Groves and Peytcheva 2008; Heggestad et al. 2015; Rindfuss et al. 2015). We therefore see some justification to assume that while the reported WRA prevalence should be treated with caution, our findings concerning the associations between WRA activity and the predictor variables are not very likely to be substantially biased by the low response rate of the survey.

As with all other available survey studies relevant to the subject of this article, the design of our study is cross-sectional in nature. The cross-sectional data allow to determine frequencies of observations as well as to establish associations between variables. The associations we found cannot be interpreted as proof that causality is actually present, since they may also result from unknown common causal variables. Even if assumed that there is a causal relationship between two variables, it cannot be said with certainty which is cause and which is effect. This would require an analysis of longitudinal data, which are, however, not available in this subject area.

It must also be noted that several aspects of WRA implementation were not—or were only roughly—covered by the GDA survey questionnaire. The work characteristics and occupational hazards accounted for when performing WRAs were defined in rather broad terms that do not allow for drawing particularly firm conclusions on the actual content of the WRAs reported. Furthermore, the questionnaire did not include any items concerning the methods used for assessing workplace risks, how the risk assessment process is organised, or the specific types of preventive measures taken. Finally, no judgement on the correctness of the risk assessments can be made from the data provided, although some doubts about the accuracy of many WRAs may arise in view of the remarkably high proportion of companies (52%) reporting that no needs for improvement measures were identified in the process. Accordingly, this study, while making a clear improvement over previous survey-based research on the subject, still provides only a rough approximation of psychosocial risk assessment practices and their comprehensiveness.

## Implications for policy and practice

In view of the fact that psychosocial risks are a highly prioritised issue in European and national OSH policies, their current status in companies’ OSH practices, especially in the conduct of WRAs, seems rather disappointing. For Germany, it must be taken into consideration, however, that psychosocial risks were put on the national OSH policy agenda several years later than in Scandinavian countries (Hansen et al. 2015; Rasmussen et al. 2011) or Great Britain (Mackay et al. 2004; Mellor et al. 2011). For example, Germany’s National Action Programme for Mental Health at Work was not launched before 2013 (Ertel 2014), whereas similar initiatives in other countries partly date back from the beginning of the previous decade or even earlier (European Commission 2011). Therefore, it may be hoped that the present results only reflect the point of departure for a broader implementation of psychosocial risk assessments in future company practices. Evidence for such a development, if actually occurring, might be provided by the third wave of the GDA survey planned for 2020.

Establishing systematic psychosocial risk management at the company level is a complex process that depends on a wide range of internal and external factors and the way those factors interact (Janetzke and Ertel 2017). Our study highlights the important role of OSH specialist advisors and OSH inspection authorities in this process, indicating that enhancing the utilisation of professional OSH experts and strengthening the advisory and control capacities of competent authorities would be beneficial for improving the current situation if these measures are accompanied by continuous efforts to further develop the conceptual approaches and skills in the area of psychosocial risk management. First steps in this direction have been taken within the framework of the Joint German Occupational Safety and Health Strategy (GDA) and its Action Programme for Mental Health at Work, which includes, among other things, a provision for additional training of all OSH inspectors, the development of standard curricula for OSH expert training in psychosocial issues and the creation of special consulting units within OSH inspectorates (Geschäftsstelle der Nationalen Arbeitsschutzkonferenz 2017). It should be considered, however, that the chances for achieving progress in psychosocial risk assessment are not simply a matter of effectively organising the OSH system but must be analysed in the wider context of socioeconomic change, competition-driven management strategies and transforming labour relations (Walters et al. 2011).

## Conclusion

As mentioned before, this study could only provide a rough picture of the current practices, drivers and barriers related to psychosocial risk assessment. To extend our knowledge about how and why companies—especially small ones—succeed (or fail) in managing these risks, improved research, either by means of in-depth case studies or survey studies using more sophisticated items, is needed. Empirical information acquired from such studies may be highly useful for providing companies with adequate and carefully targeted support in the field of psychosocial risk assessment.

## Abbreviations

CATI: Computer-assisted telephone interview
ESENER: European Survey of Enterprises on New and Emerging Risks
EU: European Union
GDA: Gemeinsame Deutsche Arbeitsschutzstrategie (“Joint German Occupational Safety and Health Strategy”)
OR: Odds ratio
OSH: Occupational safety and health
SPSS: Statistical Package for the Social Sciences
WRA: Workplace risk assessment
